# Attitudes to and Experiences of Physical Activity After Colon Cancer Diagnosis Amongst Physically Active Individuals – A Qualitative Study

**DOI:** 10.1177/10732748221119352

**Published:** 2022-09-06

**Authors:** David Renman, Karin Strigård, Richard Palmqvist, Pia Näsvall, Ulf Gunnarsson, Anette Edin-Liljegren

**Affiliations:** 1 Department of Surgical and Perioperative Sciences, Surgery, 206100 Umeå University, Umeå, Sweden; 2 Department of Medical Biosciences, Pathology, 377074 Umeå University, Umeå, Sweden; 3 Department of Epidemiology and Global Health, 174480 Umeå University, Umeå, Sweden

**Keywords:** content analysis, colon cancer, qualitative research, interview study, physical activity

## Abstract

**Background:**

Physical activity improves survival, reduces postoperative complications, and reduces the risk of developing colon cancer. It is important to maintain physical activity after receiving a diagnosis of colon cancer to improve postoperative recovery. Individuals who are physically active and diagnosed with colon cancer presumably have different motivations to maintain physical activity compared to their sedentary counterparts.

**Objective:**

Enlighten how the diagnosis of colon cancer might affect physically active individuals in their attitude and experiences towards physical activity.

**Methods:**

A qualitative study using content analysis was conducted in northern Sweden based on semi-structured telephone interviews of twenty patients diagnosed with colon cancer. All participants met the recommendations for physical activity issued by the World Health Organization.

**Results:**

Participants were between 50 and 88 years and 50% were male. Three main categories were identified: I’ll fight the cancer and come out stronger; The diagnosis makes no difference; and The diagnosis is an obstacle for physical activity. These main categories represent the ways the individuals reacted to the diagnosis of colon cancer regarding their physical activity.

**Conclusion:**

Attitudes to and experience of physical activity after colon cancer diagnosis varied from a will to increase physical activity and fight the cancer, to the diagnosis putting a stop to physical activity. It is important that healthcare professionals recommend physical activity even in already physically active individuals, to encourage continued physical activity after diagnosis of colon cancer.

## Introduction

Physical activity reduces the risk for several forms of cancer and improves survival after treatment.^1^ The benefit of physical activity in colon cancer patients is substantial since physical activity both before and after diagnosis improves survival by approximately 20%.^2,3^ In Sweden, the median age when diagnosed with colon cancer is 74 years,^4^ and risk factors include several lifestyle-related factors such as obesity, diabetes, high intake of alcoholic beverages, processed and red meat, and low intake of dietary fiber, dairy products and wholegrains.^5-7^ The colon cancer population is generally older with a more sedentary behavior and health profile than the general population.^5^ Furthermore, previous studies have shown that better preoperative physical performance may be associated with faster recovery after abdominal surgery,^8,9^ and shorter in-hospital stay after cancer surgery.^10^

The World Health Organization (WHO) recommends that all adults, also those aged 65 and above, should undertake moderate aerobic physical activity for at least 150-300 minutes per week, or vigorous aerobic physical activity for 75-150 minutes per week and muscle-strengthening activities two times per week.^11^ The proportion of the younger Swedish population adhering to these recommendations is approximately 70%. However, with rising age the proportion of persons who are physically active at least 150 minutes per week steadily declines, and among those aged 65-84 years, only 54% follow these recommendations.^12^ Northern Sweden is sparsely populated with an elderly population. Greater distance to the treating hospital has been associated with delayed diagnosis^13^ and more advanced disease.^14^ Living far from the treating hospital has also been associated with lower physical function and quality-of-life among colorectal cancer survivors.^15^

Receiving a cancer diagnosis can be life-changing not only due to the physical impact of the disease but also the mental challenges. A recent qualitative study on older patients on their attitude towards physical activity prior to colorectal cancer surgery concluded that they were generally aware of the benefits of physical activity prior to surgery but did nothing about it.^16^ Prehabilitation *i.e.*, optimization of physical status before surgery, is an emerging field in cancer research. Recent systematic reviews have shown fewer postoperative complications,^17,18^ improved functional fitness,^19^ and shorter length of stay^20^ after abdominal surgery. However, many studies suffer from differing prehabilitation schemes and endpoints, and consequently the actual effect of prehabilitation prior to abdominal surgery remains uncertain. Two qualitative studies on how patients experience prehabilitation prior to rectal cancer surgery found that patients were generally positive. Examples of increased vitality, a more positive attitude, social improvement, and a sense of purpose, structure, and control were expressed. Furthermore, taking up physical activities prior to surgery motivated them to continue exercising afterwards.^21,22^ Other qualitative studies have explored similar aspects among cancer survivors during and after adjuvant treatment, coming to the conclusion that exercise-based rehabilitation has psychological benefits, helps individuals to preserve autonomy and normal identity, and restores trust in their body.^23^

Continuation of physical activity prior to surgery is important since deterioration in physical fitness while waiting for colorectal surgery has been associated with an increase in serious postoperative complications^24^ as well as higher risk for further impairment of physical function 6 months after surgery.^25^ It is obviously a different thing to encourage a physically active patient to maintain activity level than to persuade a sedentary patient to begin physical activity while waiting for surgery. To our knowledge, no previous study has explored the views, experiences, and attitudes toward physical activity in physically active patients diagnosed with colon cancer scheduled for curative surgery. The aim of this study was to enlighten how the diagnosis of colon cancer might affect physically active individuals in their attitude and experiences towards physical activity.

## Methods

This was a qualitative study using semi-structured interviews analyzed with content analysis and is reported according to Consolidated Criteria for Reporting Qualitative Research (COREQ)^26^(Supplementary Table 1).

### Participants

**Figure 1.**
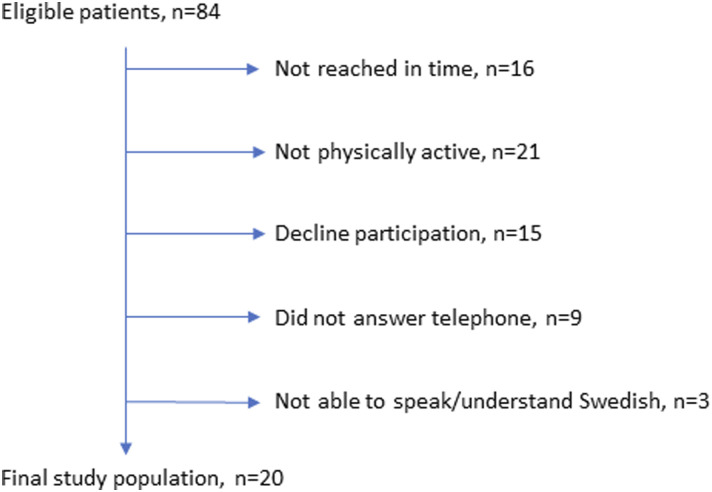
Eligible Participants and Final Study Population.

Study participants were recruited between September 2020 and November 2021 from three healthcare regions in northern Sweden: Region Västerbotten, Region Västernorrland, and Region Norrbotten. Inclusion criteria were, age > 18 years, diagnosed with colon cancer, eligible for primary surgery with curative intent (stage I-III), able to speak and understand Swedish, no neoadjuvant chemotherapeutic treatment, and self-reported physical activity level according to national recommendations from the Swedish National Board of Health and Welfare.^27^ The participants were reached by telephone by the first author. After three attempts without response, they were considered as non-responders and excluded. Participants who declined participation were for ethical reasons not asked to explain their reasons since this could increase the risk of unwillingly accepting participation in the study. This schedule for communication with the patients was approved by the ethical comitte. Exclusions are shown in the Figure 1. Participants resided varying distances from the treating hospital: mean 76.8 km, range 700m - 250 km. Distance from home to the treating hospital was estimated using Google Maps^TM^.

### Procedure

Written information about the study was sent by mail to all eligible patients after they had received their colon cancer diagnosis at the hospital. A few days later, they were contacted by telephone and informed about the study. Patients interested in participating in the study were asked, during the initial telephone contact, to complete a validated questionnaire comprising two questions regarding recreational physical and everyday exercise.^27^ Answers to the two questions were given a score, and a total score ≥11 indicated that the participant fulfilled the physical activity recommendations of the Swedish National Board of Health and Welfare. If the participant fulfilled all inclusion criteria and verbal consent had been given, a time for the interview was fixed and the participant filled in the informed consent form attached to the study information. A questionnaire regarding educational status, marital status, height, weight, country of birth, and work status was also included with the study information. One participant did not fill in this questionnaire and two others had missing information regarding educational status. These were also included in the study.

All interviews were conducted by the first author (DR), a male M.D. and PhD-student who, at the time of the study, was working as surgical resident in a county hospital in Sweden. DR had no previous experience in semi-structured interviews or qualitative research but had participated in a course in qualitative research at post-graduate level. DR had no previous contact with the participants and was not involved in their care.

### Data Collection

Semi-structured interviews were conducted over the phone and were audio-recorded. All audio recordings were transcribed word by word by a secretary. An interview guide (Table 1) was established after discussion between three of the authors (DR, KS, AEL). KS is professor of surgery with previous experience of, and supervising PhD students, in qualitative research, and AEL is an associate professor with a great deal of experience in different qualitative research methods. After the first three interviews, the authors went through the transcriptions to evaluate if the questions satisfied the aim of the study and whether the interviews were conducted satisfactorily. This resulted in the addition of an extra question regarding information of physical activity in the interview guide. The first three interviews were included in the study in their original form. Data saturation was discussed among the authors and after 15 interviews no further information emerged. There was no repeat interview, and no notes were taken during the interviews. The transcripts were not sent to the participants for their comments.

**Table 1.** Main Interview Guide. Clarifying and follow-up questions were asked when appropriate. For example, “Can You Give Example”, “Can You Elaborate?”, “What Did You Mean by That”.

Main Questions
1. What is physical activity for you?
2. Describe if your physical activity has changed after your diagnosis? If the answer was that the physical activity was changed then ask: how?
3. What prevents you or prohibits physical activity after your diagnosis? What has been most difficult? Is there anything that facilitates physical activity after diagnosis?
4. What has motivated you to do physical activity after your diagnosis?
5. What advantages are there with physical activity?
6. What disadvantages are there with physical activity?
7. How has recovery after physical activity been since your diagnosis?
8. Have you received information from the hospital regarding physical activity since your diagnosis? Is that anything you would have wanted?
9. Is there anything you want to add concerning physical activity and your diagnosis?

### Data Analysis

Qualitative content analysis with an inductive approach according to Graneheim and Lundman^28^ was performed. To obtain a sense of the whole, all transcripts were read and re-read. The entire texts were divided into shorter meaning units and further condensed (see Table 2). These condensed meaning units were further labelled with a descriptive code of the content, close to the original text with the aim of the study in mind. The codes were then abstracted and sorted into sub-categories that were further abstracted into categories and main-categories. No code was sorted into more than one sub-category. All data were coded, but after discussion between the authors, codes not related to the aim of the study were not included in the final abstraction.^29^ The first stages were conducted by DR, and codes, sub-categories, categories, and main-categories were regularly and repeatedly discussed amongst the other authors. To assure there was agreement on coding and avoid possible data loss in the coding process, in three of the interviews all stages of data analysis DR conducted together with two of the authors (KS and AEL). For data structure and analysis, Microsoft Excel was used (Microsoft Corp, Redmond, WA, USA). Table 2 shows an example of the analysis procedure. The participants did not provide feedback on the findings.

**Table 2.** Example of the Scheme of Analysis.

Meaning unit	Condensed meaning unit	Code	Sub-category	Category	Main category
The feeling is that it is serious now, sort of. I have to take this serious and not just because, what do you call it, for amusement. It sounds wrong but it is serious now. In a different way.	The feeling is that it is serious now. I have to take it serious and not just for amusement. It is serious now, in a different way.	Taking physical activity seriously	Greater determination after diagnosis	Positive effects and increased motivation for physical activity	I’ll fight this cancer and come out stronger

This study was approved by the Swedish Ethical Review Authority (Dnr 2020-01661 and 2021-00553).

## Results

### Participants

In all, 10 men and 10 women were interviewed. Age when interviewed ranged between 50 to 88 years (mean 70.4), and BMI ranged 20.8 to 32.4 kg/m^2^ (mean 25.1 kg/m^2^, missing n = 1). Distance to treating hospital ranged between 700 m and 250 km (mean 76.8 km). All participants were born in Sweden except one who had another first language. Ten of the participants were from Region Västerbotten, six from Region Västernorrland, and four from Region Norrbotten. The interviews lasted 3.8 to 28.6 minutes (mean 14.1 minutes). Six participants had completed elementary school, six had completed secondary school, and five had postsecondary education (missing n = 3). Of the participants, 16 were married, 1 in a partnership, 1 single, and 1 widow/widower (missing n = 1).

### General Views on Physical Activity

Views on what physical activity is differed between participants, some included walking, cycling, and dancing, while others cross-country skiing, running, heavyweight lifting, and step training among others. Many of the participants performed physical activity outdoors, and it was not unusual to regard working outside or in the woods as physical activity. A few participants described physical activity as doing something that includes extra effort. Several participants had pain or injury that affected their ability to perform physical activity while others expressed that their level of physical activity had fallen with age. The interviews occurred during the COVID-19 pandemic and several of the participants reported that their physical activity had been affected by the pandemic due to cancelled group training or closed fitness centers, but they still achieved the level of physical activity required for inclusion.

For many, physical activity formed a large part of their lives and was given priority. The advantages experienced of physical activity included emotional wellbeing, feeling healthy, keeping the body in shape, better sleep, being able to do more than peers, being sociable, and having fun. However, some expressed the risk for injuries and time consumption as being minor disadvantages.

Eleven categories emerged from the interviews of which three main categories were abstracted: 1. I’ll fight this cancer and come out stronger; 2. The diagnosis makes no difference; and 3. The diagnosis is an obstacle for physical activity (Table 3).

**Table 3.** Main-Categories and Their Categories.

Main category	Category
I’ll fight this cancer and come out stronger	Self-education about physical activity and cancer
	Positive effects and increased motivation for physical activity
	Being informed about physical activity is important
	Hope for the future
The diagnosis makes no difference	Lifestyle and physical activity as before
	Recovery after physical activity has not changed
The diagnosis is an obstacle for physical activity	Less or modified physical activity after diagnosis
	Physical symptoms of the cancer
	Experience of lack of time
	Worries about the time after surgery
	Disturbing thoughts about the diagnosis

### Main-Category 1: I’ll Fight This Cancer and Come out Stronger

Increased motivation and greater determination to carry on with physical activity were described. Increased motivation was explained by the will to recover after the surgery and to prevent relapse of the disease. Others were motivated by the will to continue to be physically active after surgery. The foundation for the increased motivation and positive effects of physical activity on the disease was described as an awareness of benefits from physical activity on the recovery after surgery as well as on the prognosis of the cancer. A desire to receive information from healthcare regarding physical activity, cancer, and surgery before and after the operation was expressed.

*I feel this (physical activity) is serious now. I must take it seriously - it’s not just, what do you say, for amusement. It may sound strange, but I take this thing seriously in a different way now. Put in another way, I must do it*. - Female, 79 years old.

#### Self-Education About Physical Activity and Cancer

Knowledge about the positive effects of physical activity on the disease itself as well as on postoperative recovery was described. The benefits mentioned were primarily getting prepared for surgery and rapid postoperative recovery. Some knew of the positive effects of physical activity on disease even before diagnosis, while others received this information after diagnosis.

*I’ve read about it - you can affect the cancer if you are physically active*. - Male, 69 years old.

#### Positive Effects and Increased Motivation for Physical Activity

Participants clearly described how their cancer diagnosis had had a positive effect on their attitude to physical activity as well as how physical activity was carried out. Determination to perform better, a more serious attitude toward physical activity, and a greater desire to be physically active were described. One participant described how he never skipped scheduled workouts after getting the diagnosis. Increased physical activity after diagnosis and physical activity being experienced as more fun were two of the positive effects.

Participants experienced greater motivation to perform physical activity because of their desire to cope with surgery or to improve postoperative recovery.

*Now I must be more physically active so that I am prepared for surgery. Because when you lie down in the operation theatre you don’t move at all*. - Female, 87 years old.

A wish to be physically active after surgery was also something that increased motivation. A belief that physical activity could decrease the risk of relapse of the cancer was described. Statements of increased motivation due to a belief that physical activity affects the outcome of surgery and the postoperative recovery in a beneficial way were communicated.

*What motivates me is that the better shape I’m in, the faster I heal and the better are my chances of survival… that the cancer doesn’t come back if I’m in good shape and feeling well*. - Female, 79 years old.

#### Being Informed About Physical Activity is Important

Participants described the desire to receive information from healthcare professionals regarding physical activity. Opinions on how such information should be provided differed; some wanted written information, others preferred information by mouth, while others wanted information to be available online.

*I would like a brochure clearly saying how to do it and why […] what type of physical activity you should do and some tips regarding how to get into better shape before and after the operation*-Female, 79 years old.

#### Hope for the Future

Participants were interviewed between the times of diagnosis and planned surgery, and it was evident that participants focused on their operation and the future. The period between diagnosis and surgery was mostly experienced as waiting, and questions regarding the time prior to surgery were sometimes answered by phrases concerning the time after surgery. All participants were scheduled for surgery with curative intent, and they described feelings of hope for the future and the will to rapidly return to their ordinary physical activity after surgery. Feelings of hopefulness were experienced among the participants waiting for surgery.

*I know I’ll come back and that my fitness will eventually return*. - Female, 80 years old.

### Main-Category 2: The Diagnosis Makes No Difference

Experience of unchanged lifestyle or physical activity after the diagnosis was described by participants. They kept living their life as before the diagnosis without altering it too much. Primarily, those who hadn’t experienced any symptoms from their cancer were more likely to express that they lived their life as before. However, also participants that had experienced different symptoms did not alter their degree of physical activity and were still being active as before the diagnosis.

#### Lifestyle and Physical Activity as Before

Participants said they could carry out the same physical activities as before their diagnosis, even a few with symptoms.

*Towards the end I was soiling my pants and had to wear a diaper […] but I continued with my physical activities. Yesterday I was out walking as usual*. - Male, 76 years old.

Those who didn’t alter their physical activity usually had no symptoms or symptoms relieved by symptomatic treatment. These individuals continued with physical activity as usual after diagnosis.

*I don’t think my physical activity has changed since I got the diagnosis. I still take walks and push on as usual*. - Female, 79 years old.

Life consists of more than physical activity and expressions of no changes after diagnosis were described. Most of these participants had been symptomless for the time after diagnosis which of course facilitates not changing anything. One participant actively tried not to think about the diagnosis while another said that he hadn’t had the time to think about the diagnosis.

*No, nothing has changed. I’ve had this cancer in my abdomen for a couple of years without knowing about it. I’m not changing anything just because I have a diagnosis*. - Male, 77 years old.

#### Recovery After Physical Activity Has Not Changed

Information that recovery after physical activity wasn’t altered after receiving the diagnosis was collected from the interviews. In some patients with low-intensity training, this was probably because any change in recovery before diagnosis was hardly noticeable. Others still recovered rapidly regardless of the diagnosis.

*There is no difference compared to before. It’s the same. I don’t conduct hard physical activity so I don’t see any change in recovery*. - Male, 73 years old.

### Main-Category 3: The Diagnosis Is an Obstacle for Physical Activity

Sometimes the ability, possibility, or motivation for physical activity became lower and some experienced it hard to be physically active after diagnosis. Participants with physical or mental symptoms found it more difficult to be physically active. However, for some patients with symptoms, physical activity remained unchanged or increased. For some participants, mostly those living in rural areas, time-consuming visits to healthcare facilities for examinations and blood sampling interfered with their physical activity. Some were worried about being able to carry on with physical activity after surgery as well as emotional problems that affected physical activity.

#### Less or Modified Physical Activity After Diagnosis

Participants described less physical activity after diagnosis, the reasons for this being physical symptoms, feeling down, and being more careful. Other participants described that their physical activity had changed after diagnosis due to symptoms and time spent in visits and examinations at the hospital. Some described how they changed from one form of physical activity to another to fit in better with their life, while others changed the time of day they exercised because symptoms varied over the day.

*I’ve had problems with my bowels and not been able to do physical activity when I’d like. I’ve had to wait a while before it gets better during the morning […] so I’ve had to skip physical activity in the morning* - Female, 64 years old.

*For practical reasons I’ve had to reduce physical activity because of all the hospital appointments that come in the way*. - Male, 76 years old.

#### Physical Symptoms of the Cancer

Anemia was the usual cause of cancer symptoms, but once anemic patients had received treatment their symptoms resolved. Other symptoms described were abdominal pain, changed bowel habits, problems with sleep, muscle pain, and vertigo.

*In fact, I’ve had symptoms for nine months, but I didn’t understand what it was. My blood value dropped, and I wasn’t aware of that. I thought something was wrong with my heart, but eventually it turned out that my blood value was extremely low*. - Male, 72 years old.

#### Experience of Lack of Time

Some participants described that hospital visits and other examinations after the cancer diagnosis was made were time-consuming. One participant said that after diagnosis, the time previously spent on physical activity was spent on the disease.

*Before I spent time on physical activity, but now that time is spent dealing with the disease; visiting the hospital and healthcare center, making phone calls to doctors and nurses etc*. - Male, 75 years old.

For others the time between diagnosis and planned surgery was experienced as short. There was so much to do during that time that physical activity was affected.

*It’s only a few days since the diagnosis and already I’ve had several hospital visits for examinations. I haven’t had time to think about physical activity*. - Female, 61 years old.

#### Worries About the Time After Surgery

Participants described their concern over not to be able to be physically active after the operation, or were uncertain about recovery after surgery, or expected pain after surgery.

*I’m worried I won’t be able to do as much physical activity after surgery. I think there will be a healing period when I won’t be able to be active, but I’ll do what I can*. - Female, 70 years old.

Other participants expressed the importance of being careful during the postoperative period, realizing they can’t carry on as before surgery.

*It is major surgery. As you know, I’ve had surgery before but not as major as this, so I know I’ll not be in the same shape after. You must realize that you can’t carry on in the same way as before*. - Female, 87 years old.

#### Disturbing Thoughts About the Diagnosis

For many, receiving a cancer diagnosis is life changing. One patient said that the diagnosis came out of nowhere and described it as a shock. One person expressed that he would have stopped doing physical activity if the cancer meant death, others felt that having a disease with good prognosis was essential if one was to continue exercising.

*My general practitioner sent a referral for colonoscopy, and the 14*^*th*^ *December I found out it showed a tumor in the colon. It came as a shock.*- Female, 51 years old.

Emotions and thoughts after the diagnosis prevented normal physical activity for some of the participants. One expressed that there had been several changes in his life since the diagnosis.

*It’s all the thoughts going on that prevent physical activity - constant thoughts like: That’s it! what’s going to happen? Then again; That’s it! Will I manage? Will it end up well? Will I survive the operation?* - Male, 76 years old.

## Discussion

Three main-categories emerged from content analysis of the interviews in this qualitative study on physically active individuals diagnosed with stage I-III colon cancer eligible for curative surgery. Attitudes and experiences towards physical activity after receiving the diagnosis of colon cancer varied. Some expressed the will to increase physical activity, some experienced the diagnosis as an obstacle to continued physical activity, while for some physical activity continued as usual. These are novel results of a previous unstudied population in qualitative research, physically active colon cancer patients, that emphasizes the importance for healthcare professionals to support colon cancer patients to maintain and increase physical activity.

### Experiences and Attitudes Towards Physical Activity

The result of our study with varying attitudes and experiences somewhat contrast previous studies on attitude towards preoperative exercise, where the views has been generally positive amongst patients with colorectal cancer.^21,22^ Our study population differ from those in previous research since all participants fulfilled the WHO guidelines for physical activity. In this study, some individuals expressed a negative attitude to physical activity after receiving the diagnosis. In a previous study from Karlsson *et al* those already physically active expressed openness towards preoperative exercise, while those with a negative attitude to physical activity showed little interest in conducting exercises.^16^ Theirs and our results agree regarding postoperative recovery being a major motivation for exercise, and participants in Karlsson’s study also described time-consuming examinations as being an obstacle to physical activity.^16^ Burke *et al* described predominantly positive experiences regarding preoperative physical activity described in the themes: increased vitality, a positive attitude, enhanced social connections, and a strong sense of purpose.^21^ The attitudes and experiences of the participants in the present study were not as generally positive, as seen by the rather widespread picture represented in our three main-categories. One possible explanation for this could be that Burke recruited participants from a study on a preoperative exercise program, and the views of the participants could thus have been affected.^21^ Participants in the present study were already physically active prior to diagnosis, but experiences of and attitudes towards physical activity after diagnosis varied. Some participants described increased motivation and greater determination whereas others experienced the diagnosis as an obstacle to physical activity. This variation in experiences and actions to physical activity could be due to patients reacting differently to their colon cancer diagnosis. In a meta-synthesis of experiences after receiving a colorectal cancer diagnosis, two key attitudes emerged: an optimistic one *e.g.*, achieving coherence and spending more time with the family; and a pessimistic *e.g.*, negative emotions and worrying about the treatment and future.^30^ In yet another study, reactions to the diagnosis varied from participants who were unaffected emotionally to those with more pronounced emotional reactions.^31^

### Theoretical Framework

Receiving a cancer diagnosis is associated with psychological stress. In a recent qualitative study on patients diagnosed with colorectal cancer undergoing surgery, all 24 participants experienced distress during treatment.^32^ One way of understanding the role physical activity has for physically active individuals in coping with the disease may be explained by the theory of stress, appraisal, and coping, by Lazarus and Folkmann.^33^ They describe dealing with psychological stress as an ongoing relationship between the person and the environment, with the two affecting each other.^33^ The relationship is defined by two processes: 1) appraisal, the cognitive process for evaluating what coping process is required; and 2) coping defined as “the cognitive and behavioral effects made to master, tolerate or reduce external and internal demands and conflicts among them”.^33^ There are three primary appraisal patterns: irrelevant, benign-positive, and stressful. According to this theory, when a person encounters stress, one of these three primary appraisal patterns is “chosen”. If the person decides that something is at stake, a secondary appraisal is made where coping options are evaluated to best deal with the situation. In this model, the individual can use two different coping strategies, problem-based or emotional-based. Problem-based coping is used when the person feels that she/he has control over the situation, and emotional-based coping is used when there is no feeling of control or ability to affect the outcome of the problem. The participants in this study coped with the diagnosis in different ways and physical activity was likewise affected differently. An example of problem-based coping is seen in the main-category *“I’ll fight this cancer and come out stronger”* and emotional-based is seen in the main-category *“The diagnosis is an obstacle for physical activity”*. The views expressed in the main-category *“The diagnosis makes no difference”* could be explained by the diagnosis not being perceived as stressful by these participants and therefore a secondary appraisal was not made. However, it could also be argued that this main category is an example of problem-based coping since physical activity was carried out as before despite the circumstances.

### Physical Activity as Coping Strategy

Our results imply that patients diagnosed with colon cancer can use physical activity as a coping strategy. In a recent qualitative study on colon cancer survivors, the authors concluded that physical activity was an integral part of coping after treatment as well.^34^ Regardless of whether physical activity is used as a coping strategy or not, it is important that physically active individuals stay active between diagnosis and surgery, as this can reduce postoperative complications and improve recovery.^9,17,19,35^ Even though many participants said they had increased physical activity and motivation, many described physical activity as more difficult after receiving the diagnosis. It is important that healthcare professionals address this issue and encourage physical activity both before and after surgery. Physically active patients should be easily motivated to continue physical activity if they know there is lot to lose if they stop being physically active before surgery.

### Other Factors Affecting Physical Activity

Living far from the treating hospital is associated with delayed diagnosis and more advanced disease.^13,14^ In our study, the distance from home to the treating hospital ranged between 700m and 250 km. Northern Sweden is sparsely populated, and for patients living in rural areas, visits to hospital can be time-consuming. Participants described how time required for their disease was an obstacle to exercising after diagnosis. In sparsely populated areas, logistics concerning cancer diagnosis, examinations, and treatment can easily reduce the patients’ quality of life. This also applies to physically active individuals and their ability to keep exercising and maintain their quality of life in the period leading up to surgery. This study not only included participants from rural areas and eight of the participants were living within ten km from the treating hospital. We believe that this increases the transferability^28^ of the study not only to the northern parts of Sweden but to all of Sweden and similar countries such as other countries in Scandinavia.

The most common physical symptoms were direct or indirect consequences of anemia. Although these patients had difficulty in performing physical activity before receiving treatment, a considerable proportion continued exercising. All participants with anemia received treatment with either iron supplementation or blood transfusion. Around 25% of patients with colon cancer have anemia when diagnosed.^36^ Aerobic physical activity is affected even by mild anemia with hemoglobin counts <13.9 g/dL.^37^ Physically active individuals strain their aerobic system more than sedentary individuals and therefore anemia is likely to be felt earlier. It is important that healthcare professionals react when hearing individuals with mild anemia expressing fatigue when exercising, and that treatment for anemia is initiated early to facilitate physical activity.

### Strengths and Limitations

In this study, the participants knew that the interviews concerned physical activity and they were all physically active individuals. It is possible that responses to the questions were affected by this. However, the aim of the study was to interview physically active individuals and therefore this was difficult to avoid. The risk of such effect may have been reduced by using telephone interviews, since the respondent may feel less obliged to please the interviewer. The interviews lasted, on average, around 14 minutes, but the shortest was just under four minutes. This interview was analyzed and judged to contain information relevant to the aim and was included. Since data saturation was already reached after 15 patients, and five more participants were recruited after that, the short interview was not considered a problem when interpreting the data and removing it could have affected the credibility of the study. Because of the geographic distances involved and the COVID-19 pandemic with social restrictions, all interviews were conducted by telephone despite the fact that most qualitative research uses face-to-face interviews. However, previous qualitative studies using telephone interviews have concluded that it is as good as or even better than face-to-face interviews, and are more popular with the participants.^38,39^

To increase the credibility and transferability^28^ of the study, the cohort had a wide distribution of sex and age, with different operating hospitals and regions. The validated questionnaire used in assessing information regarding physical activity was also chosen to increase credibility. The questionnaire and our interview questions did not distinguish between aerobic and muscle-strengthening physical activity. Therefore, we can not draw conclusions on differences between different types of physical activity which is a limitation of this study. The variety of the authors’ backgrounds, with different genders, professions, and ages is important for the credibility of the study.^28^ The authors contributed with different perspectives and preconceptions of both physical activity, colorectal cancer, and qualitative research. Throughout the entire analysis procedure, the interpretation and findings were repeatedly discussed, and consensus was achieved. This repeated discussion was intended not only to increase the credibility of the study but also the dependability, since it increases the possibility to maintain consistency during data interpretation.^28^ Only one researcher read all of the transcripts of the interviews which is a limitation of this study and decreases the credibility since potential codes could have been missed. In order to avoid this potential loss, three of transcripts were read by additional two of the authors (KS and AEL) and consensus on the coding process was achieved. The use of citations and an example of the scheme of analysis (Table 1) is seen as something that increases the credibility of a qualitative study.^28^

## Conclusions

In this study on physically active individuals, the attitude and experience towards physical activity after colon cancer diagnosis varied from the will to increase physical activity and fight the cancer to the diagnosis being an obstacle to continued physical activity. These are novel results that highlights that also physically active individuals may face difficulties continuing physical activity after a colon cancer diagnosis. Future studies on physical activity and pre-habilitation prior to cancer surgery should include physically active individuals to broaden their potential results and not miss an important category of patients. Our results imply that physical activity may be used as a coping strategy for the patient when faced with colon cancer. In the clinical setting, it is important that healthcare professionals actively asks about physical activity and advise physically active individuals to continue and to explain how physical activity in the period between colon cancer diagnosis and surgery hastens recovery and improves outcomes.

## Supplemental Material

Supplemental Material - Attitudes to and Experiences of Physical Activity After Colon Cancer Diagnosis Amongst Physically Active Individuals – A Qualitative Study Click here for additional data file. Supplemental material for Attitudes to and Experiences of Physical Activity After Colon Cancer Diagnosis Amongst Physically Active Individuals – A Qualitative Study by David Renman, Karin Strigård, Richard Palmqvist, Pia Näsvall, Ulf Gunnarsson, and Anette Edin-Liljegren in Cancer Control

## Appendix

### Abbreviations

BMI: Body mass index.
COREQ: Consolidated Criteria for Reporting Qualitative Research.
MD: Medical doctor.
WHO: World Health Organization.
